# Characterization of Bacteriophage cd2, a Siphophage Infecting Carnobacterium divergens and a Representative Species of a New Genus of Phage

**DOI:** 10.1128/spectrum.00973-23

**Published:** 2023-07-17

**Authors:** Angelle P. Britton, Kaitlyn A. Visser, Véronique M. A. Ongenae, Peipei Zhang, Heather Wassink, Thomas A. Doerksen, Catherine A. Welke, Karlene H. Lynch, Marco J. van Belkum, Jonathan J. Dennis, Xianqin Yang, Dennis Claessen, Ariane Briegel, Leah A. Martin-Visscher

**Affiliations:** a Department of Chemistry, The King’s University, Edmonton, Alberta, Canada; b Molecular Biotechnology, Institute of Biology, Leiden University, Leiden, the Netherlands; c Centre for Microbial Cell Biology, Leiden University, Leiden, the Netherlands; d Agriculture and Agri-Food Canada, Lacombe, Alberta, Canada; e Department of Biological Sciences, University of Alberta, Edmonton, Alberta, Canada; f Department of Chemistry, University of Alberta, Edmonton, Alberta, Canada; National Research Council of Italy

**Keywords:** carnobacteria, *Carnobacterium divergens*, bacteriophage, siphophage, B3 morphotype, subtyping

## Abstract

Carnobacterium divergens is frequently isolated from natural environments and is a predominant species found in refrigerated foods, particularly meat, seafood, and dairy. While there is substantial interest in using *C. divergens* as biopreservatives and/or probiotics, some strains are known to be fish pathogens, and the uncontrolled growth of *C. divergens* has been associated with food spoilage. Bacteriophages offer a selective approach to identify and control the growth of bacteria; however, to date, few phages targeting *C. divergens* have been reported. In this study, we characterize bacteriophage cd2, which we recently isolated from minced beef. A detailed host range study reveals that phage cd2 infects certain phylogenetic groups of *C. divergens.* This phage has a latent period of 60 min and a burst size of ~28 PFU/infected cell. The phage was found to be acid and heat sensitive, with a complete loss of phage activity when stored at pH 2 or heated to 60°C. Electron microscopy shows that phage cd2 is a siphophage, and while it shares the B3 morphotype with a unique cluster of *Listeria* and *Enterococcus* phages, a comparison of genomes reveals that phage cd2 comprises a new genus of phage, which we have termed as *Carnodivirus*.

**IMPORTANCE** Currently, very little is known about phages that infect carnobacteria, an important genus of lactic acid bacteria with both beneficial and detrimental effects in the food and aquaculture industries. This report provides a detailed characterization of phage cd2, a novel siphophage that targets Carnobacterium divergens, and sets the groundwork for understanding the biology of these phages and their potential use in the detection and biocontrol of *C. divergens* isolates.

## INTRODUCTION

Carnobacterium divergens and Carnobacterium maltaromaticum are lactic acid bacteria (LAB) commonly found in refrigerated foods, meat and dairy products, and vacuum packaged meat (1–3). The role of these bacteria within food products is complex. They are known to produce branched alcohols and aldehydes which have been associated with food spoilage (2, 4), although it has also been shown that these metabolic by-products have low sensory impact on food quality (5). On the other hand, carnobacteria may play a key role in enhancing the shelf life of packaged meat products via the production of bacteriocins (ribosomally synthesized antimicrobial peptides) and organic acids that inhibit the growth of other food spoilage organisms (6–8). Carnobacteria are also a key component of the natural microbiota in the gastrointestinal tract of healthy fish (3, 9, 10). Although some strains of carnobacteria are known fish pathogens (2, 3, 9, 10), there is substantial interest in exploring the potential of *C. divergens* as a probiotic in fish hatcheries to enhance the immunity and survival of fish larvae (11).

Because carnobacteria have the potential to impart both positive and negative impact within the food industry and aquaculture, it is desirable to have rapid and economical methods to identify and, if necessary, selectively eliminate specific carnobacteria isolates. One potential approach is the use of bacteriophages (phages) for phage typing and as biocontrol agents. Phages are viruses that can be classified as either lytic or temperate (lysogenic) according to their life cycle. After adsorption and insertion of genetic material into the host, lytic phages immediately hijack the host’s cellular machinery to assemble new phage particles and then trigger cell lysis by phage encoded proteins, resulting in the release of phage progeny and concomitant death of the host. In contrast, temperate phages are maintained as a prophage within the host cell, either by integrating directly with the host’s genome or as extrachromosomal DNA. After exposure to specific environmental stimuli, temperate phages are induced into the lytic phase of their life cycle (12). Phages can vary in their specificity of hosts, with some capable of infecting a single strain (narrow host range) or multiple hosts from even different genera (broad host range). This variability in host range enables the selection of phages to infect a single host lineage or an entire group of host lineages, which has led to biotechnological applications such as phage typing to rapidly identify bacterial isolates, and phage therapy to selectively eliminate a particular bacterium. Indeed, several phage-derived products are commercially available and used by the food industry to combat known food pathogens such as Escherichia coli, *Listeria*, Salmonella, and *Shigella* spp. (13, 14).

However, the presence of phages can also result in undesirable consequences, particularly in the food industry and the production of fermented foods. In these settings, phage infection can decimate bacterial starter cultures, resulting in poor quality food products and substantial economic losses (13, 15–17). For this reason, phages that target LAB, particularly *Lactococcus* and *Lactobacillus* spp., have been extensively studied, with the hope that understanding their biology and genetic diversity will enable the development of antiphage strategies (15, 18). In contrast, very little is known about the phages that target carnobacteria as only a few phages targeting this class of LAB have been reported. Bacteriophage cd1, first described in 1997, is a myophage and exhibits lytic activity against a limited number of *C. divergens* strains (19). In 2004, it was reported that phage LP65, a Lactobacillus plantarum myophage, could infect certain strains of carnobacteria (20). Recently, we have isolated three new phages (phages cd2, cd3, and cd4) from *C. divergens* and reported their sequences (21). Analysis of the phage genomes indicated that they were likely siphophages with limited homology to several known *Enterococcus* and *Listeria* phages within the genera *Saphexavirus* and *Homburgvirus*, respectively. The aim of the present study was to characterize phage cd2 and describe its morphology, growth kinetics, stability, and host range. This work is key to understanding the biology and potential applications of phages that infect carnobacteria, and sheds light on a new genus of tailed phages.

## RESULTS AND DISCUSSION

### Phage isolation, plaque morphology, and optimal multiplicity of infection.

**FIG 1:**
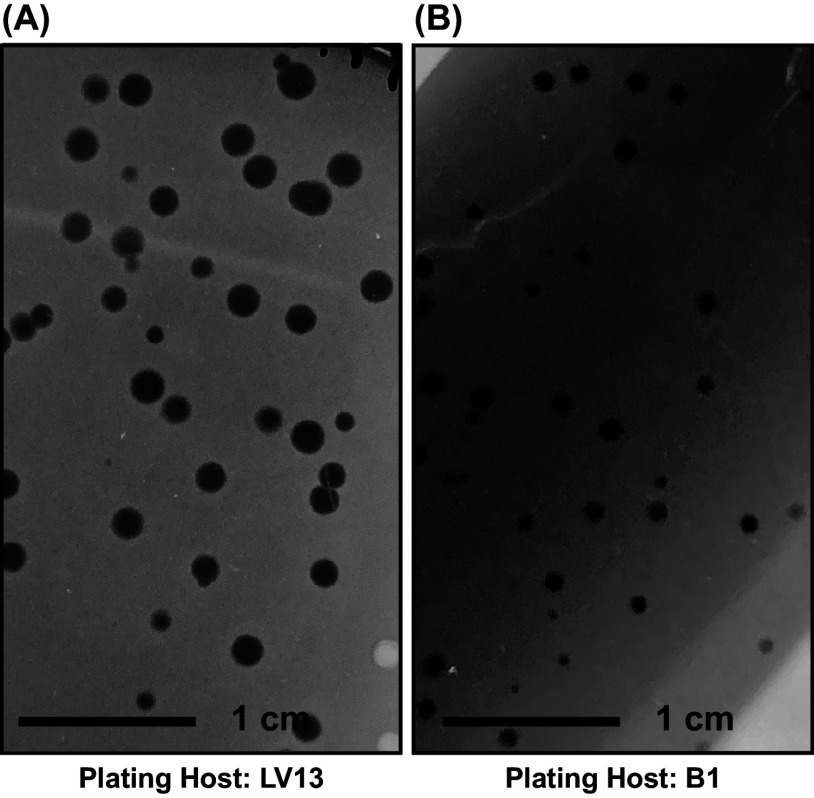
Morphology of phage cd2 plaques when plated on *C. divergens* LV13 (A) and B1 (B). Larger, clearer plaques were observed when LV13 was used as the plating host.

Phage cd2 was isolated from a sample of ground beef and, after three successive rounds of single-plaque isolation, propagated to a titer of ~10^9^ PFU/mL using *C. divergens* LV13 and B1 as hosts. Plaque morphology differed depending on the plating host. With LV13, clear plaques with sharp edges and an average diameter of ~1 mm were observed (Fig. 1A). When plated against B1, both clear and turbid pinpoint plaques (<0.5 mm) were observed (Fig. 1B). Because the genes encoded on the phage cd2 genome lack sequence homology to known integrases, it was first thought that phage cd2 is a lytic phage, and the differences in plaque morphology were due to poor adsorption of the phage onto the host, a longer latency period and/or a small burst size, or a low rate of diffusion of phage in the agar overlay (22). However, when PCR using phage cd2 specific primers was performed on genomic DNA from host cells isolated from the center of both clear and turbid plaques, positive PCR results were obtained (details provided in the supplemental material). This suggests that phage cd2 is not virulent, but is capable of lysogeny or pseudo-lysogeny. Further characterization of the numerous hypothetical proteins encoded in phage cd2’s genome may reveal the proteins required for lysogeny. Because larger and clearer plaques were observed when phage cd2 was plated against LV13, this strain was used as the plating host for subsequent experiments. The optimal multiplicity of infection (MOI) for phage cd2 with host LV13 was found to be 1.0 (Fig. S1).

### Phage morphology and phylogenetic analyses.

**FIG 2:**
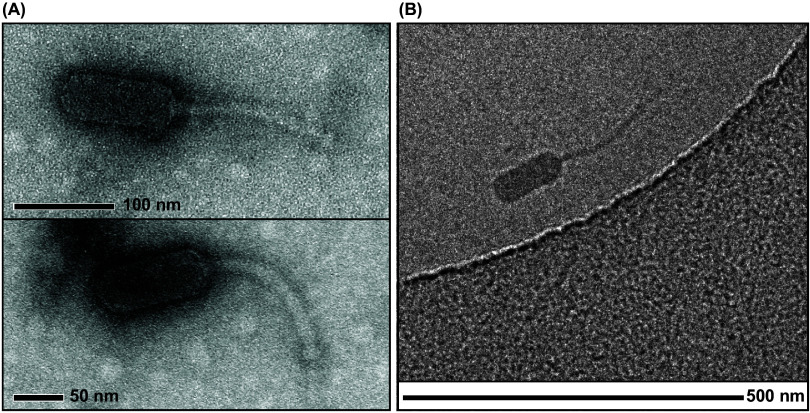
Electron microscopy reveals that phage cd2 is a siphophage (B3 morphotype). (A) TEM micrographs of phage cd2 stained with uranyl acetate and obtained at 140,000 × magnification. (B) Cryo-EM micrograph of phage cd2. Phage cd2 has an elongated capsid measuring 112 ± 6 nm × 45 ± 3 nm in width (*n* = 10), and a long, noncontractile, flexible tail measuring 164 ± 4 nm × 9.6 ± 0.9 nm (*n* = 5).

The morphology of phage cd2 was evaluated by both transmission electron microscopy (TEM) (Fig. 2A) and cryo-electron microscopy (cryo-EM) (Fig. 2B). Phage cd2 has an elongated capsid (length 112 ± 6 nm; width 45 ± 3 nm, *n* = 10) and a long, noncontractile tail (length 164 ± 4 nm; width 9.6 ± 0.9 nm, *n* = 5). No tail fibers were visible in any of the micrographs. These results confirm our earlier prediction, based on genomic analysis (21), that phage cd2 is a siphophage and belongs to the class *Caudoviricetes.* Aside from our work, there have only been two prior reports of phages known to infect carnobacteria, and in both cases these phages (bacteriophage cd1 and Lactobacillus plantarum bacteriophage LP65) were shown to be myophages (19, 20). As we previously reported, a BLASTN search of phage cd2’s genome did not find closely related phages (21). Taken together, these results reveal that phage cd2 is morphologically and genetically distinct from phage cd1 and LP65, and represents a unique group of phages isolated from and infecting carnobacteria.

**FIG 3:**
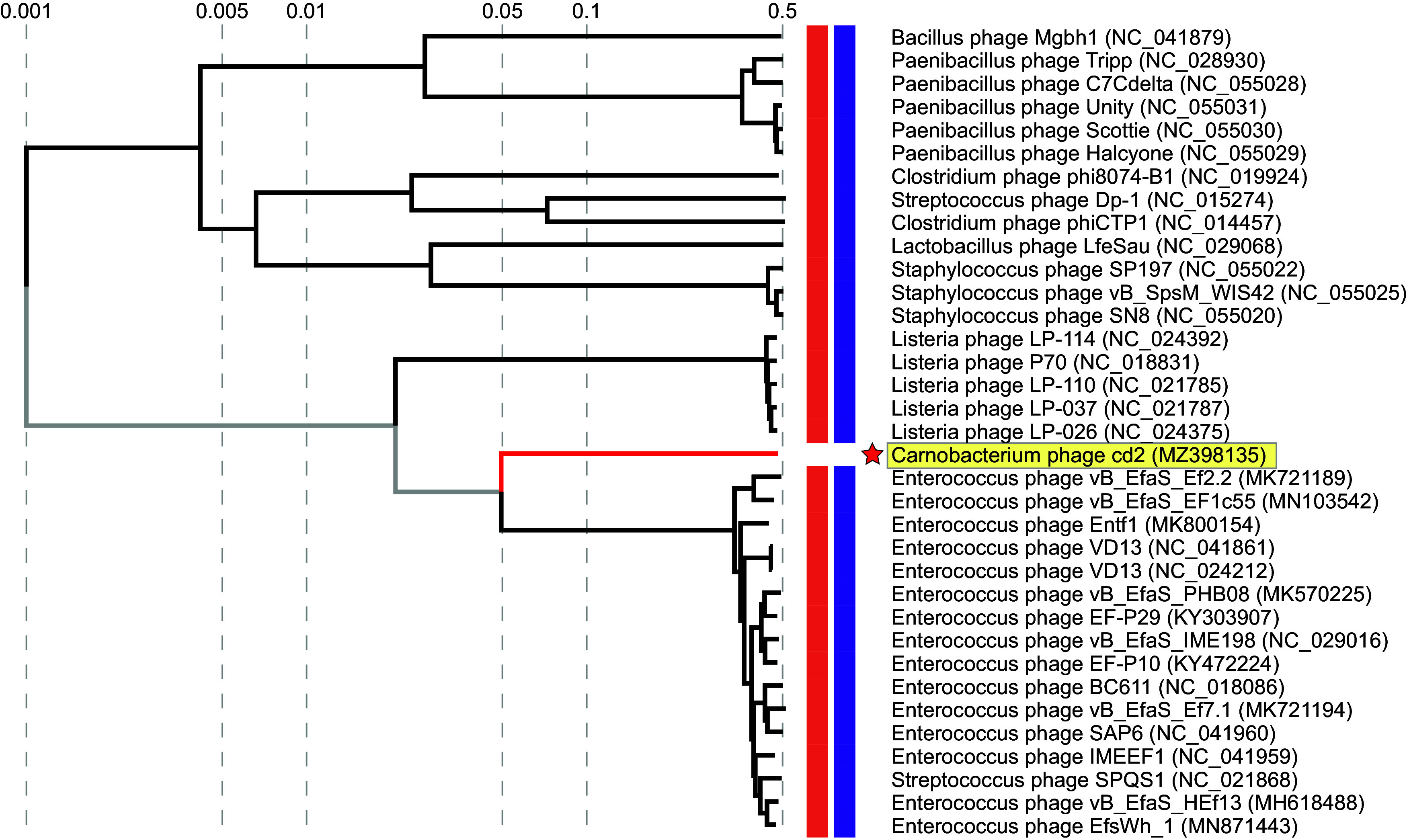
Proteomic tree for phage cd2. Only phages displaying a genomic similarity score greater than 0.01 (S_G_ > 0.01) are included. The colored bars beside each phage indicate phage morphology (orange = siphophage) and host group (purple = *Bacillota*). The position of phage cd2 is indicated by a red star and yellow box. Accession numbers for phage genomes are provided in the figure. Figure generated using ViPTree (30).

**FIG 4:**
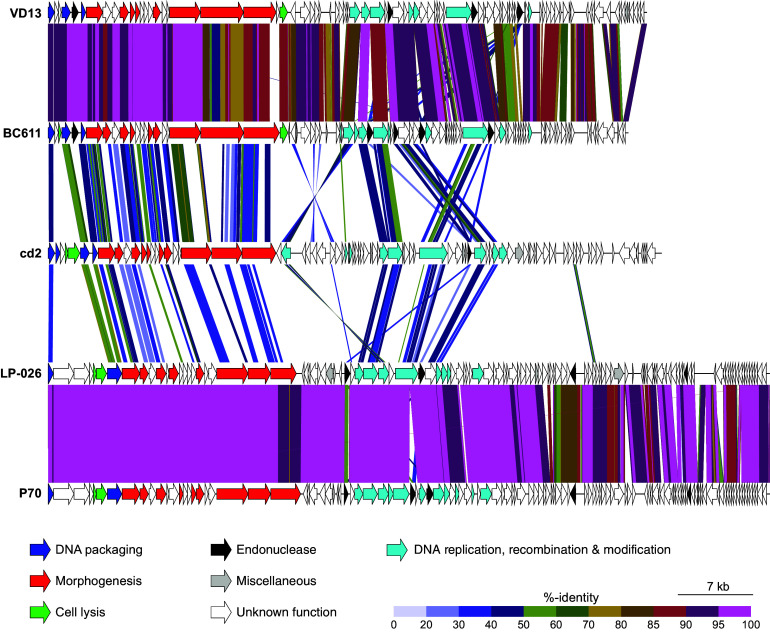
Genomic alignment of phage cd2 with *Enterococcus* phages BC611, VD13, and *Listeria* phages LP-026 and P70. Predicted genes are indicated with arrows and color coded by putative function. The colored vertical blocks between genomes indicate the level of nucleotide similarity. Genomes were reordered to begin at the terminase small subunit. Accession numbers and references for the phage genomes used in this analysis are provided in Table S4. Alignment generated using ViPTree (30).

The elongated capsid displayed by phage cd2 falls within the B3 morphotype, which is characterized by a prolate capsid with length-to-width ratio of 2.5 to 5.5 (23–25). While relatively uncommon (24), this same morphotype has also been reported for a unique group of *Listeria* phages belonging to the genus *Homburgvirus* (26–28), and *Enterococcus* phages belonging to the genus *Saphexavirus* (26, 29). Phylogenetic analysis has shown that these two genera of phages cluster together and share nine unique orthologs that encode for putative proteins involved in phage morphology and DNA replication. Their genomes also encode for between one and five HNH endonucleases, where one is located downstream of DNA polymerase (26). Interestingly, when we analyzed phage cd2’s genome with ViPTree (30), the resulting proteomic tree identified these two genera of phages as the most closely related to phage cd2 (Fig. 3). A genomic alignment of phage cd2 with representative phages from each of these genera reveals that while there is a low degree of nucleotide similarity between the phages, they all display a shared gene synteny (Fig. 4). Notably, phage cd2’s genome encodes for putative lysis enzymes within the DNA packaging module (consistent with the *Listeria* phages in genus *Homburgvirus*) and a putative HNH endonuclease located downstream of DNA polymerase. Thus, it is likely that phage cd2 also resides within this unique orthocluster of phages, characterized by the B3 morphotype and a conserved gene synteny. Using VIRIDIC (31), we further examined the intergenomic similarity between phage cd2 and these phages (Fig. S2). As the total genome sequence identity was well below 70%, phage cd2 can be classified in a new genus (32). The International Committee on Taxonomy of Viruses (ICTV) has approved our proposal for the creation of a new genus called *Carnodivirus*, with phage cd2 being a representative species. In accordance with ICTV nomenclature rules, phage cd2’s official binomial name is *Carnodiviru*s *cd2*.

### Growth kinetics: adsorption and one-step growth curve.

**FIG 5:**
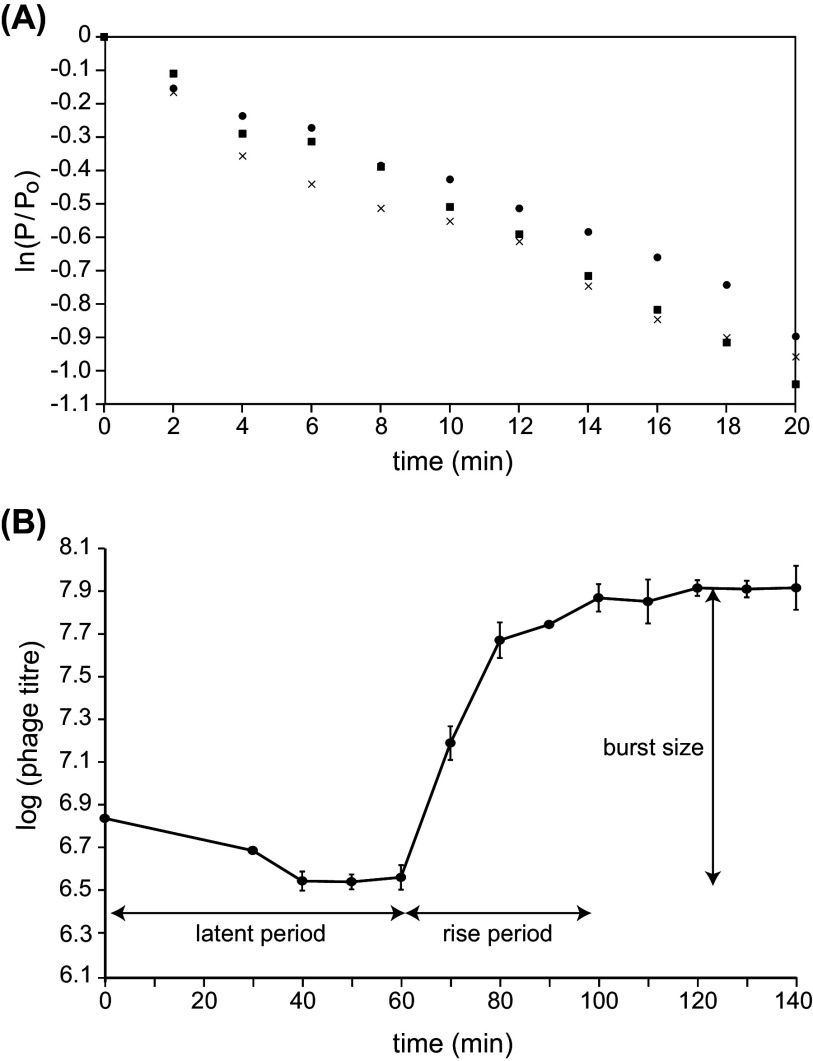
Growth kinetics for phage cd2: adsorption curve (A) and one-step growth curve (B). In panel A, different symbols represent different replicate assays. For each assay, the adsorption rate was calculated by dividing the absolute value of the slope of the adsorption curve by the bacterial density used in the assay. In panel B, the data point at *t* = 0 represents the total phage titer initially added to the *C. divergens* LV13 culture. The first titer measurement was collected 30 min later, following the 10 min incubation period and centrifugation steps to collect infected cells. Data represents the average result ± standard deviation for three biological replicates.

An adsorption experiment was performed to determine phage cd2’s adsorption rate with host LV13. Phage cd2 was added to a culture of LV13 and the amount of unadsorbed phage was quantified every 2 min, for 20 min. As shown in Fig. 5A, the relative concentration of phage cd2 decreased over the course of the experiment. Taking into account the initial bacterial density used in each replicate assay, the adsorption rate for phage cd2 was calculated to be 2.26 × 10^−10 ^mL/min.

Because phage cd2 has a low adsorption rate, two key modifications to the one-step growth curve experiment were made (33). First, to increase the number of infected cells, the phage-host cell mixture was incubated for 10 min, rather than 5 min. Second, to remove unadsorbed phage, the mixture was centrifuged and after rinsing, only the pellet, containing phage-infected cells, was used in the subsequent dilution steps and for enumeration of phage titer. The one-step growth curve (Fig. 5B), shows that when LV13 is used as the host strain, phage cd2 has a latent period of ~60 min. The burst size of phage cd2 was determined to be ~28 PFU/infected cell, which is smaller than the reported burst sizes for several of the enterococcal phages (which range from 60 to 350 PFU/infected cell) that share a similar morphology to cd2 (34–38). On the other hand, phage cd2’s burst size is comparable to what has been reported for several *Lactobacillus* phages (39–41).

### Stability of phage cd2.

**FIG 6:**
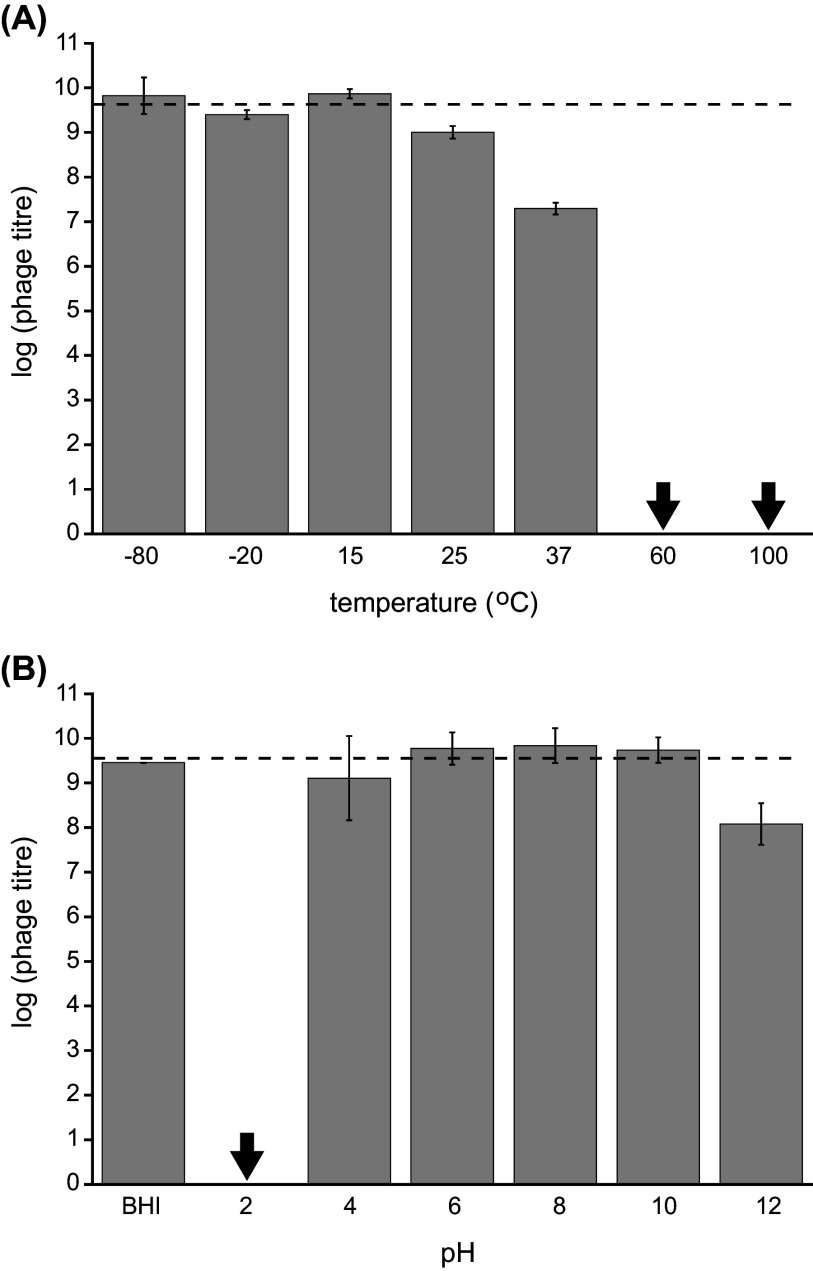
Stability of phage cd2 at various temperatures (A) and pH conditions (B) using *C. divergens* LV13 as plating host. For the temperature study, phage samples were incubated for 4 days at temperatures of −80 to 37°C, and for 15 min at temperatures of 60 and 100°C. For the pH study, phage was incubated for 2 h. The pH of the BHI control was 7.4. Black arrows indicate that no plaques were observed on any plates, including undiluted samples. The dashed line represents the initial log_10_ phage titer used in the treatments. Data represents the average log_10_ titer ± standard deviation for two biological replicates, plated in triplicate.

Given that carnobacteria are often associated with refrigerated foods and meat products, we examined the stability of phage cd2 under various thermal and pH conditions using strain LV13 as plating host (Fig. 6). Phage titers remained the highest when the phage was stored at temperatures below 25°C for 4 days, indicating that phage cd2 is stable at cool temperatures. When stored at 25°C for 4 days, a slight reduction in phage titer was observed. Phage survival was dramatically reduced at higher temperatures: after 4 days at 37°C, phage titer decreased by a factor of 100, and when heated to 60°C or higher for just 15 min, no viable phage was detected (Fig. 6A). Phage cd2 was unaffected at pH conditions ranging from 4 to 10, but either highly basic or acidic conditions decreased phage survival. At pH 2, phage cd2 was completely deactivated (Fig. 6B).

Recently, it was demonstrated that the production of organic acids by *Carnobacterium* spp. can reduce the pH of meat juice medium from 5.9 to 5.2 and this is one of the key factors in their ability to inhibit the growth of both Gram-positive and Gram-negative food-spoilage bacteria (6). However, these conditions also enable carnobacteria to become the dominant microbiota in vacuum packaged meats, which has also been implicated in food spoilage (2, 8). Our results suggest that phage cd2 could serve as a useful biocontrol agent for *C. divergens* strains in refrigerated meat products because phage cd2 survives at both the temperatures and pH conditions under which carnobacteria thrive. On the other hand, if phage infection is not desired, our results indicate that a simple heat treatment can be used to destroy the phage.

### Host range studies.

Phage cd2 was tested against a panel of *C. divergens* and *C. maltaromaticum* strains, all of which have previously been isolated from beef, pork, or fish products. The results of the spot test (Table 1) show that phage cd2 displayed lytic activity against several *C. divergens* strains but was unable to infect any of the *C. maltaromaticum* strains tested. Aside from LV13, the *C. divergens* isolates used in this study have previously been separated into eight different groups (IV to XI) according to phylogenetic analysis of their genomes (6). Interestingly, the susceptibility of *C. divergens* strains to phage cd2 was correlated with their grouping. Strains belonging to groups V to VII all showed sensitivity to phage cd2 as zones of inhibition were observed. For strains in groups V and VI, the spots were clear, while the spot against strain C13 (group VII) was turbid. When PCR was performed, using phage cd2 specific primers and genomic DNA from C13 cells isolated from these plaques, no positive results were obtained (details provided in supplemental material). We also tested the supernatant from a phage-free culture of strain B1 against the group V to VII isolates and no zones of inhibition were detected. This confirms that the sensitivity of isolates in group V to VII is due to phage cd2, and not some other component in the phage lysate, such as an unknown bacteriocin produced by the host strain. For strains belonging to groups IV and VIII to XI, no zones of clearing were detected, revealing that these strains are resistant to phage cd2. These results show that while phage cd2 has a species-specific host range, it can effectively discriminate between different phylogenetic groups of *C. divergens* strains. This property of phage cd2 has potential applications for the rapid and facile subtyping of *C. divergens* isolates.

**TABLE 1:** Host range of phage cd2 by spot tests and efficiency of plating (EOP)

Host strain	Phylogenetic group^*a*^	Spot test^*b*^	EOP (%)^*c*^	Reference
Carnobacterium divergens				
LV13		+	100; M	50
A10	IV	−		51
B1	V	+	58 ± 0.01; P	51
B6	V	+	70 ± 0.06; P	51
C5	V	+	60 ± 0.06; P	51
C7	V	+	60 ± 0.02; P	51
C10	V	+	61 ± 0.03; P	51
C14	V	+	63 ± 0.04; P	51
B7	VI	+	8 ± 0.01; M	51
B8	VI	+	18 ± 0.0; M	51
C6	VI	+	18 ± 0.01; M	51
C16	VI	+	18 ± 0.02; M	51
C13	VII	(+)	0	51
B3	VIII	−		51
C1	VIII	−		51
C12	VIII	−		51
C17	VIII	−		51
C8	IX	−		51
B5	X	−		51
C3	X	−		51
C4	X	−		51
C9	X	−		51
C11	X	−		51
C15	X	−		51
A2	XI	−		51
A4	XI	−		51
A8	XI	−		51
A12	XI	−		51
Carnobacterium maltaromaticum				
LV17B	NA	−		50
A9b-	NA	−		52
C2	NA	−		53
UAL307	NA	−		54
UAL26	NA	−		55

a *C. divergens* isolates were divided into phylogenetic groups by core genome alignment as previously described (51).

b +, clear zone of inhibition; (+) turbid zone of inhibition; −, no sensitivity to phage.

c Results represent the average EOP ± standard deviation for three biological replicates. M, clear plaques with average diameter of ~1mm; P; clear and turbid pinpoint plaques (<0.5 mm).

To further explore the ability of phage cd2 to infect sensitive strains belonging to groups V to VII, we performed an efficiency of plating (EOP) experiment (Table 1). Using strain LV13 as our reference (EOP of 100%), we found that strains within group V displayed EOPs of ~60% (range 58% to 70%) and produced plaques that were consistently smaller than those on LV13. Strains within group VI displayed EOPs of ~15% (range 8% to 18%) but had plaques of similar size to LV13. Interestingly, the EOP of strain C13 (group VII) was 0%, irrespective of the initial phage titer used in the assay. This suggests that the interaction between phage cd2 and C13 does not result in a productive phage infection.

**FIG 7:**
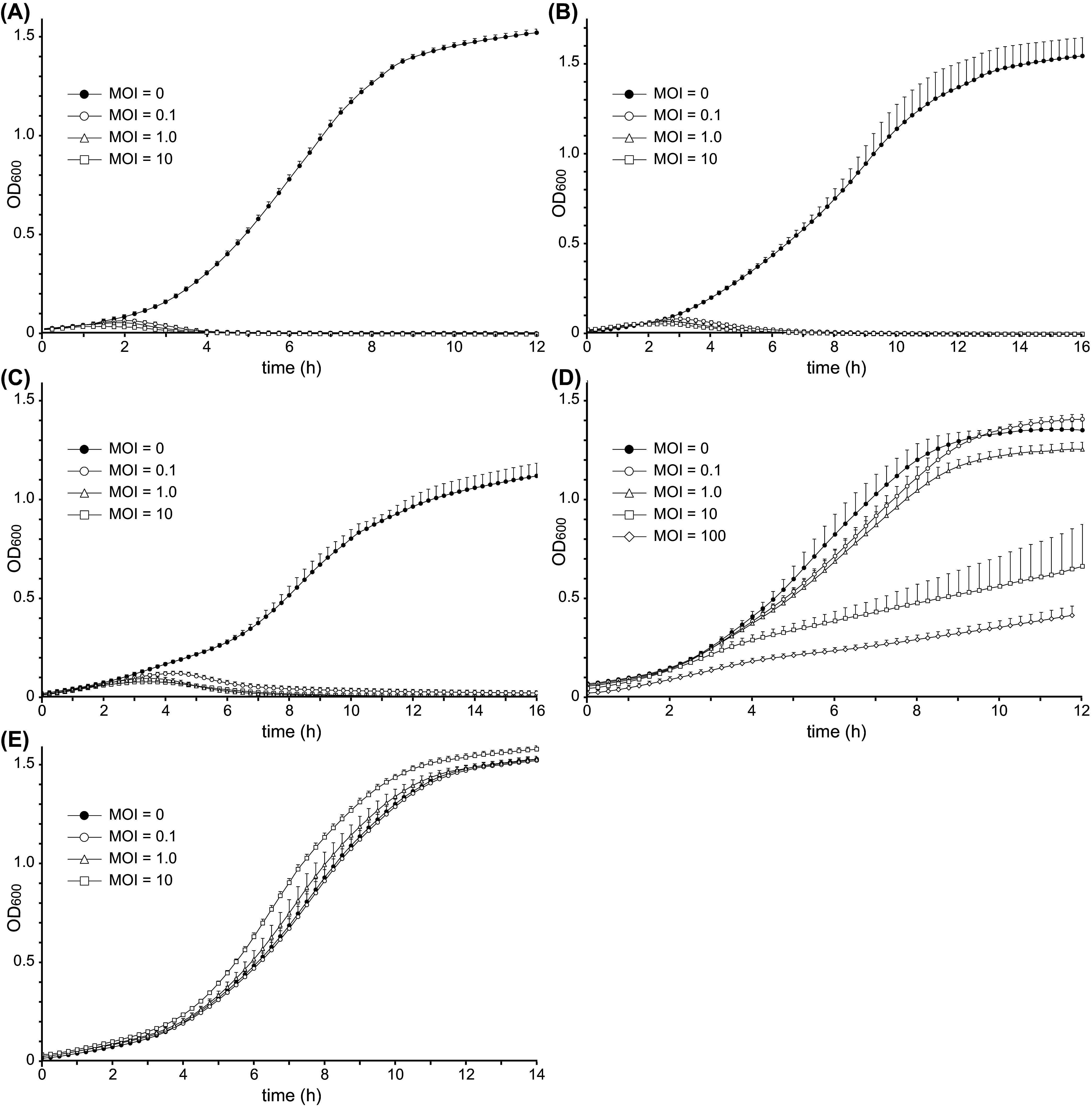
The effect of phage cd2 on the growth of *C. divergens* strains LV13 (A), B1 (B), C16 (C), C13 (D), and C3 (E). Strains B1, C16, and C13 are phage sensitive and belong to phylogenetic groups V, VI, and VII, respectively. Strain C3 is phage resistant and belongs to phylogenetic group X. Phage was added at different MOIs, as indicated by the legends. Data represents the average result for at least five technical replicates. Error bars represent the standard deviation and for clarity, only the positive error bars are plotted.

Because we observed different EOPs among the sensitive strains, we examined the impact of phage cd2, at differing concentrations, on the growth curves of various *C. divergens* strains (Fig. 7). The results show for that for strains LV13 (Fig. 7A), B1 (group V, Fig. 7B), and C16 (group VI, Fig. 7C), phage cd2 clearly inhibited bacterial growth at all MOIs tested. The time needed to reach inhibition (OD_600_ <0.1) was fastest for LV13 and longer for both B1 and C16, which is consistent with our finding that these latter two strains have reduced EOPs relative to LV13. The observed differences in EOP may indicate that strains B1 and C16 are partially resistant to phage cd2; however, it is also known that bacterial growth rate can impact phage kinetics (adsorption, latent period, and burst size) and hence phage population growth (42). Our results show that in the absence of phage cd2, strains B1 and C16 both grew slower than LV13 under the conditions tested, and therefore this may also be responsible for the reduced EOP demonstrated by these strains. Further studies of these strains with phage cd2, to compare adsorption rates, one-step growth curves, and the impact of growth conditions on phage fecundity, will help elucidate the different interactions between phage cd2 and these sensitive strains. Nevertheless, our results clearly demonstrate that phage cd2 can effectively inhibit the growth of these strains, even when added at an MOI of 0.1. As expected, phage cd2 had no impact on the growth of C3, a resistant strain belonging to group X, at any of the MOIs tested (Fig. 7E).

In the case of strain C13, our results reveal that the impact of phage cd2 on bacterial growth is concentration dependent (Fig. 7D). When phage was added at an MOI of 0.1, no detectable effect on bacterial growth was observed, while an MOI of 1.0 resulted in a slight reduction of bacterial growth. On the other hand, when phage was added at an MOI of 10 and 100, the growth rate of C13 was dramatically reduced. These results are consistent with our earlier findings from both the spot and EOP assays, and suggest that the sensitivity of strain C13 to phage cd2 at high MOIs is due to a “lysis from without” mechanism, while at low MOIs, there is a nonproductive phage infection (43).

As mentioned above, our host range studies showed that phage cd2 effectively discriminated between different phylogenetic groups of *C. divergens*. Because the genomes for all these isolates, aside from LV13, have previously been reported, we were interested in comparing the genomes of the sensitive and resistant strains to identify putative phage receptors. To this end, we first sequenced the genome of LV13 and then performed a phylogenetic analysis on all the *C. divergens* isolates used in this study (complete details for both steps are provided in Table S1). First, our results showed that strain LV13 shares the largest orthologous average nucleotide identity with isolates in group VI (Table S2). Indeed, all the strains within group VI were sensitive to phage cd2, and while they had lower EOPs relative to LV13, similar plaque morphologies were observed for LV13 and the group VI isolates. The results from our core single-nucleotide polymorphism analysis revealed a significant degree of genetic variation between the phage-sensitive and phage-resistant isolates (Table S3). As such, it was not possible to highlight key genetic differences in core genomes that could be linked to sensitivity or resistance to phage cd2. Additional studies will be needed to identify potential phage receptors. One such approach is to generate phage-resistant mutants from isolates in groups V and VI, followed by whole-genome sequencing and comparing the genomes of the mutant strains to the wild-type, sensitive strains.

### Conclusion.

While phages infecting *Lactococcus* and *Lactobacillus* spp. have been extensively studied, virtually nothing is known about phages that infect carnobacteria. This genus of lactic acid bacteria has a complex role within the food and aquaculture industries, exhibiting both positive and negative impacts. Our studies with phage cd2 represent the first, detailed account of a phage that targets *C. divergens*. We have shown that phage cd2 is a siphophage, capable of distinguishing between different phylogenetic groups of *C. divergens* strains. Analysis of phage cd2’s physicochemical properties identifies readily controllable parameters to maintain or destroy phage viability. As additional phages targeting carnobacteria are discovered and characterized, we will better understand the diversity of these phages and their potential applications in industrially relevant settings for the detection and biocontrol of both desirable and problematic *Carnobacterium* spp.

## MATERIALS AND METHODS

### Bacterial strains, media, and growth conditions.

The bacterial strains used in this study are listed in Table 1. All strains were grown in brain heart infusion (BHI) media (Bacto) and incubated at 25°C. Solid media contained 1.5% (wt/vol) agar, while soft agar contained 0.5% to 0.75% (wt/vol) agar. When media was used for phage studies, 2 mM CaCl_2_ was added. Strains were maintained as frozen stocks at −78°C with 20% glycerol. Dilutions of both phage and bacteria were done by addition of BHI broth. Filter sterilization was completed using 0.22 μm syringe driven filters. Unless otherwise stated, all centrifugation steps were performed at 9,000 × *g*, 5 min, 4°C.

### Phage purification.

Bacteriophage cd2 was initially isolated from the culture broth of minced beef, as previously described (21). Details are provided in the supplemental material. Phage titer, expressed in PFU/mL, was determined using the double agar overlay method (44). High titer phage stocks (~10^9^ PFU/mL) were prepared using three consecutive rounds of single-plaque isolation, using either *C. divergens* LV13 or *C. divergens* B1 as the host strain. Phage lysate was stored at 4°C.

### Determination of optimal MOI.

Phage lysate (10^9^ PFU/mL) was serially diluted to prepare a series of phage solutions. Equal volumes of the phage solutions and a culture of LV13 (either 10^7^ CFU/mL or 10^8^ CFU/mL) were mixed, corresponding to MOIs of 0.0001, 0.001, 0.01, 0.1, 1, 10, and 100. The mixtures were incubated for 10 min at 25°C, centrifuged, and the pellet was resuspended in 1.5 mL of fresh media. The samples were incubated for 4 h at 25°C, after which phage titer was determined using the double agar overlay method using LV13 as the plating host. The sample providing the highest resulting titer was considered the optimal MOI. Data represents the average of three technical replicates.

### Electron microscopy.

Phage micrographs were obtained using cryo-EM and TEM. For cryo-EM, the phage sample was first concentrated by adding 1 mL of precipitation buffer (20% PEG8000, 250 mM NaCl) to 5 mL of a high titer phage sample. The mixture was incubated overnight at 4°C, centrifuged (3,000 × *g*, 1 h, 4°C), and the supernatant discarded. Three μL of the pelleted sample was added to a glow-discharged R2/2 200 mesh holey carbon EM grid (Quantifoil) for 30 s and the sample was blotted for 1 s, after which the grid was plunged in liquid ethane using a Leica GP automated freeze-plunger. The vitrified samples were observed using 626 side-entry cryo-holder (Gatan) inside a 120 kV Talos Cryo-EM with a Lab6 electron source and Ceta detector at the Netherlands Center for Nanoscopy (NeCEN, Leiden). For TEM, a high titer phage sample was incubated for 5 min on a carbon-coated copper grid and then stained for 30 s with saturated uranyl acetate. Micrographs were taken with the assistance of the University of Alberta Department of Biological Sciences Advanced Microscopy Facility using a Philips/FEI (Morgagni) transmission electron microscope with charge-coupled device camera at 140,000× magnification.

### Determination of adsorption rate.

An adsorption study (45) was performed to determine the adsorption rate for phage cd2. Briefly, 1 mL of phage lysate (~5 × 10^5^ PFU/mL) was added to 9 mL of LV13 culture (OD_600_~0.2), and the mixture was incubated at 25°C with gentle shaking. Every 2 min, for 20 min, a 100 μL sample was removed and mixed vigorously with 900 μL of fresh BHI containing 30 μL chloroform. The number of free phage in each sample was determined using the double agar overlay method, with LV13 as the plating host. For each time point, phage titer (P) was determined and used to calculate ln(P/P_o_), where P_o_ was the initial phage titer used in the experiment, and these values were plotted against time (t). The adsorption constant (k) was determined by dividing the absolute value of the slope of this adsorption curve by the bacterial density used in the experiment. Data represents the average result for three biological replicates.

### One-step growth curve.

The one-step growth experiment was performed as described by Kropinski (33), with slight modifications. A total of 100 μL of phage suspension (10^9^ PFU/mL) was mixed with 9.9 mL of a mid-log-phase culture of LV13, at an MOI of ~0.01, which was considered the start of the infection (*t* = 0). After incubating the mixture for 10 min at 25°C, a 1 mL aliquot was removed, centrifuged (3,500 × *g*, 5 min, RT), and the supernatant containing unadsorbed phage was discarded. The pellet was resuspended in 1 mL of fresh BHI broth, centrifuged, and again resuspended in 1 mL of fresh media. Then, 100 μL of this solution was transferred to 9.9 mL of fresh BHI broth (Flask A) and several 10-fold serial dilutions were prepared. To quantify any remaining unadsorbed phage, a 1 mL sample from flask A was filter sterilized and 100 μL of this sample was enumerated with the double agar overlay assay. Starting at 30 min postinfection and continuing every 10 min up to 140 min, a 100 μL aliquot from the appropriate dilution flask was removed and enumerated with the double agar overlay method, using LV13 as the plating host. Plates were incubated for 18 h at 25°C after which plaques were counted and used to determine phage titer. For each time point, the log_10_ titer was plotted against time. Data represent the average result ± standard deviation for three biological replicates. The latent time and burst size were calculated from the plotted curve, as described by Kropinski (33).

### Stability studies.

The thermal stability of phage cd2 was determined by incubating phage lysate at temperatures of −80, −20, 15, 25, and 37°C for 4 days, and at 60°C and 100°C for 15 min. The phage samples were then serially diluted and enumerated using the double agar overlay method using LV13 as the plating host. To determine the effect of pH on phage viability, 100 μL of phage lysate was mixed with 900 μL of pH-adjusted BHI media (pH values ranging from 2 to 12), where the pH of the media was modified by addition of HCl or NaOH and the final concentration of media components was kept constant. Regular BHI broth, with a pH of 7.4, was used as a control. The samples were incubated at 25°C for 2 h, then serially diluted and phage titer was determined using the double agar overlay method using LV13 as the plating host. For both thermal and pH stability studies, two biological replicates were performed, and plated in triplicate. Results are expressed as log_10_ titer ± standard deviation.

### Host range assays.

The host range of phage cd2 was examined by the spot test, where 10 μL of phage lysate (~10^9^ PFU/mL) was spotted onto an agar plate that had been overlaid with 5 mL of soft agar containing 100 μL of an overnight culture of one of *Carnobacterium* strains to be tested (see Table 1). Plates were incubated overnight and then inspected for zones of clearing. For strains that showed susceptibility to phage cd2, an EOP test was also performed (46). In brief, 100 μL of appropriately diluted phage lysate was mixed with 5 mL of soft agar containing 100 μL of an overnight culture of the host strain, and then overlaid on a plate of solid media. Following incubation for 16 to 18 h at 25°C, plaques were counted, and phage titer determined. EOP was calculated as the ratio of the phage titer when tested against the host strain relative to the phage titer when tested against LV13. EOPs are reported as a percentage and represent the average EOP ± standard deviation for three biological replicates. LV13 is able to produce the bacteriocin divergicin A (47), which may inhibit other *C. divergens* strains, and hence be a cofounding factor for the host range test. Therefore, only phage stocks that had been propagated and isolated from strain B1 were used for host range studies since B1 does not harbor bacteriocin-producing genes (6).

### Growth curves of phage-infected *C. divergens* strains.

Growth curves depicting the effect of phage cd2 at various MOIs (0, 0.1, 1, 10) on the growth of four phage-sensitive *C. divergens* strains (LV13, B1, C16, and C13) and one phage-insensitive *C. divergens* strain (C3) were constructed using a microplate assay. Overnight cultures of the bacterial strains were subinoculated into 50 mL of fresh BHI broth containing 2 mM CaCl_2_ and incubated at 25°C. When the cultures reached early exponential growth phase (~10^8^ CFU/mL), 100 μL of culture was transferred to the wells of a 96-well microtiter plate containing 100 μL of phage cd2 solution at titers of 0, 10^7^, 10^8^, and 10^9^ PFU/mL, corresponding to MOIs of 0, 0.1, 1, and 10, respectively. For the assay with strain C13, an additional MOI of 100 was tested. Microplates were sealed with breathable strips, placed in a microplate reader (TECAN Spark) and incubated at 25°C. Every 15 min the plates were gently shaken after which the OD_600_ of each well was measured. Data were collected for either 12 or 16 h, depending on the strain. For each time point, the mean and standard deviation for each sample (at least five technical replicates) was calculated and plotted against time to generate the growth curve.

### Phylogenetic analysis.

VipTree (30) was used to construct a proteomic tree by uploading phage cd2’s genome and using default settings. VipTree was further used to generate a genomic alignment, based on tBLASTx results, of phage cd2’s genome and selected reference genomes that had similarity scores greater than 0.05 (S_G_ >0.05). For the reference viral genomes, VipTree retrieved gene positions and annotations from NCBI flat files, whereas for phage cd2, gene positions were predicted using the GeneMarkS algorithm (48) within VipTree. These results were nearly identical to our previously reported analysis of phage cd2’s genome (21), in which we used PHANNOTATE v1.5.0 (49) for gene prediction and annotation. In the resulting genomic alignment image, gene functions were manually color coded, according to the annotations within the NCBI flat files for all the genomes used in the alignment. VIRIDIC (31) was then used to probe the intergenomic similarities between phage cd2 and the phages for which VipTree had reported S_G_ >0.05. The genomes of interest were saved into a single fasta file, uploaded to VIRIDIC WEB, and analyzed using default settings, including 95% and 70% as the threshold settings for the demarcation of new species and genus, respectively.

## References

[1] Rieder G, Krisch L, Fischer H, Kaufmann M, Maringer A, Wessler S. 2012. *Carnobacterium divergens* - a dominating bacterium of pork meat juice. FEMS Microbiol Lett 332:122–130. doi:10.1111/j.1574-6968.2012.02584.x. 22537055

[2] Leisner JJ, Laursen BG, Prévost H, Drider D, Dalgaard P. 2007. *Carnobacterium*: positive and negative effects in the environment and in foods. FEMS Microbiol Rev 31:592–613. doi:10.1111/j.1574-6976.2007.00080.x. 17696886 PMC2040187

[3] Hammes WP, Hertel C. 2006. The genera *Lactobacillus* and *Carnobacterium*, p 320–403. *In* Dworkin M, Falkow S, Rosenberg E, Schleifer K-H, Stackebrandt E (ed), The prokaryotes. Springer US, New York, NY.

[4] Laursen BG, Leisner JJ, Dalgaard P. 2006. *Carnobacterium* species: effect of metabolic activity and interaction with *Brochothrix thermosphacta* on sensory characteristics of modified atmosphere packed shrimp. J Agric Food Chem 54:3604–3611. doi:10.1021/jf053017f. 19127732

[5] Casaburi A, Nasi A, Ferrocino I, Di Monaco R, Mauriello G, Villani F, Ercolini D. 2011. Spoilage-related activity of *Carnobacterium maltaromaticum* strains in air-stored and vacuum-packed meat. Appl Environ Microbiol 77:7382–7393. doi:10.1128/AEM.05304-11. 21784913 PMC3194841

[6] Zhang P, Gänzle M, Yang X. 2019. Complementary antibacterial effects of bacteriocins and organic acids as revealed by comparative analysis of *Carnobacterium* spp. from meat. Appl Environ Microbiol 85:e01227-19. doi:10.1128/AEM.01227-19. 31399404 PMC6805084

[7] Mills S, Ross RP, Hill C. 2017. Bacteriocins and bacteriophage; a narrow-minded approach to food and gut microbiology. FEMS Microbiol Rev 41:S129–S153. doi:10.1093/femsre/fux022. 28830091

[8] Laursen BG, Bay L, Cleenwerck I, Vancanneyt M, Swings J, Dalgaard P, Leisner JJ. 2005. *Carnobacterium divergens* and *Carnobacterium maltaromaticum* as spoilers or protective cultures in meat and seafood: phenotypic and genotypic characterization. Syst Appl Microbiol 28:151–164. doi:10.1016/j.syapm.2004.12.001. 15830808

[9] Ringø E, Gatesoupe F-J. 1998. Lactic acid bacteria in fish: a review. Aquaculture 160:177–203. doi:10.1016/S0044-8486(97)00299-8.

[10] Ringø E, Hoseinifar SH, Ghosh K, Doan HV, Beck BR, Song SK. 2018. Lactic acid bacteria in finfish - an update. Front Microbiol 9:1818. doi:10.3389/fmicb.2018.01818. 30147679 PMC6096003

[11] Garcés ME, Olivera NL, Fernández M, Riva Rossi C, Sequeiros C. 2020. Antimicrobial activity of bacteriocin-producing *Carnobacterium* spp. isolated from healthy Patagonian trout and their potential for use in aquaculture. Aquac Res 51:4602–4612. doi:10.1111/are.14806.

[12] Guttman B, Raya R, Kutter E. 2005. Basic phage biology, p 29–66. *In* Kutter E, Sulakvelidze A (ed), Bacteriophages: biology and applications. CRC Press, Boca Raton, FL.

[13] Połaska M, Sokołowska B. 2019. Bacteriophages—a new hope or a huge problem in the food industry. AIMS Microbiol 5:324–346. doi:10.3934/microbiol.2019.4.324. 31915746 PMC6946638

[14] Li J, Zhao F, Zhan W, Li Z, Zou L, Zhao Q. 2022. Challenges for the application of bacteriophages as effective antibacterial agents in the food industry. J Sci Food Agric 102:461–471. doi:10.1002/jsfa.11505. 34487550

[15] Garneau JE, Moineau S. 2011. Bacteriophages of lactic acid bacteria and their impact on milk fermentations. Microb Cell Fact 10:S20. doi:10.1186/1475-2859-10-S1-S20. 21995802 PMC3231927

[16] Mahony J, van Sinderen D. 2014. Current taxonomy of phages infecting lactic acid bacteria. Front Microbiol 5:7. doi:10.3389/fmicb.2014.00007. 24478767 PMC3900856

[17] Kot W, Neve H, Heller KJ, Vogensen FK. 2014. Bacteriophages of *Leuconostoc, Oenococcus, and Weissella*. Front Microbiol 5:186. doi:10.3389/fmicb.2014.00186. 24817864 PMC4009412

[18] Labrie SJ, Samson JE, Moineau S. 2010. Bacteriophage resistance mechanisms. Nat Rev Microbiol 8:317–327. doi:10.1038/nrmicro2315. 20348932

[19] Manchester LN. 1997. Characterization of a bacteriophage for *Carnobacterium divergens* NCFB 2763 by host specificity and electron microscopy. Lett Appl Microbiol 25:401–404. doi:10.1111/j.1472-765x.1997.tb00005.x. 9449854

[20] Chibani-Chennoufi S, Dillmann M-L, Marvin-Guy L, Rami-Shojaei S, Brüssow H. 2004. *Lactobacillus plantarum* bacteriophage LP65: a new member of the SPO1-like genus of the family *Myoviridae*. J Bacteriol 186:7069–7083. doi:10.1128/JB.186.21.7069-7083.2004. 15489418 PMC523202

[21] Zhang P, Britton AP, Visser KA, Welke CA, Wassink H, Prins E, Yang X, Martin-Visscher LA. 2021. Genome sequences of bacteriophages cd2, cd3, and cd4, which specifically target *Carnobacterium divergens*. Microbiol Resour Announc 10:e00636-21. doi:10.1128/MRA.00636-21. 34435863 PMC8388549

[22] Abedon ST, Yin J. 2009. Bacteriophage plaques: theory and analysis, p 161–174. *In* Clokie MRJ, Kropinski AM (ed), Bacteriophages: methods and protocols, volume 1: isolation, characterization, and interactions. Humana Press, Totowa, NJ. 10.1007/978-1-60327-164-6_17 19066821

[23] Ackermann H-W, DuBow MS. 1987. Viruses of prokaryotes: general properties of bacteriophages. CRC Press, Boca Raton, FL.

[24] Ackermann H-W. 2001. Frequency of morphological phage descriptions in the year 2000. Arch Virol 146:843–857. doi:10.1007/s007050170120. 11448025

[25] Ackermann HW, Eisenstark A. 1974. The present state of phage taxonomy. Intervirology 3:201–219. doi:10.1159/000149758. 4461697

[26] Denes T, Vongkamjan K, Ackermann H-W, Moreno Switt AI, Wiedmann M, den Bakker HC. 2014. Comparative genomic and morphological analyses of *Listeria* phages isolated from farm environments. Appl Environ Microbiol 80:4616–4625. doi:10.1128/AEM.00720-14. 24837381 PMC4148797

[27] Peters TL, Hudson LK, Song Y, Denes TG. 2019. Complete genome sequences of two *Listeria* phages of the genus *Pecentumvirus*. Microbiol Resour Announc 8:e01229-19. doi:10.1128/MRA.01229-19. 31727716 PMC6856282

[28] Schmuki MM, Erne D, Loessner MJ, Klumpp J. 2012. Bacteriophage P70: unique morphology and unrelatedness to other *Listeria* bacteriophages. J Virol 86:13099–13102. doi:10.1128/JVI.02350-12. 22993158 PMC3497695

[29] Di Lallo G, Falconi M, Iacovelli F, Frezza D, D'Addabbo P. 2021. Analysis of four new *Enterococcus faecalis* phages and modeling of a hyaluronidase catalytic domain from *Saphexavirus*. Phage 2:131–141. doi:10.1089/phage.2021.0003. 36161247 PMC9041502

[30] Nishimura Y, Yoshida T, Kuronishi M, Uehara H, Ogata H, Goto S. 2017. ViPTree: the viral proteomic tree server. Bioinformatics 33:2379–2380. doi:10.1093/bioinformatics/btx157. 28379287

[31] Moraru C, Varsani A, Kropinski AM. 2020. VIRIDIC-a novel tool to calculate the intergenomic similarities of prokaryote-infecting viruses. Viruses 12:1268. doi:10.3390/v12111268. 33172115 PMC7694805

[32] Turner D, Kropinski AM, Adriaenssens EM. 2021. A roadmap for genome-based phage taxonomy. Viruses 13:506. doi:10.3390/v13030506. 33803862 PMC8003253

[33] Kropinski AM. 2018. Practical advice on the one-step growth curve, p 41–47. *In* Clokie MRJ, Kropinski AM, Lavigne R (ed), Bacteriophages: methods and protocols, volume 3. Springer New York, New York, NY.

[34] Cheng M, Liang J, Zhang Y, Hu L, Gong P, Cai R, Zhang L, Zhang H, Ge J, Ji Y, Guo Z, Feng X, Sun C, Yang Y, Lei L, Han W, Gu J. 2017. The bacteriophage EF-P29 efficiently protects against lethal vancomycin-resistant *Enterococcus faecalis* and alleviates gut microbiota imbalance in a murine bacteremia model. Front Microbiol 8:837. doi:10.3389/fmicb.2017.00837. 28536572 PMC5423268

[35] Tkachev PV, Pchelin IM, Azarov DV, Gorshkov AN, Shamova OV, Dmitriev AV, Goncharov AE. 2022. Two novel lytic bacteriophages infecting *Enterococcus* spp. are promising candidates for targeted antibacterial therapy. Viruses 14:831. doi:10.3390/v14040831. 35458561 PMC9030284

[36] Zhang W, Mi Z, Yin X, Fan H, An X, Zhang Z, Chen J, Tong Y. 2013. Characterization of *Enterococcus faecalis* phage IME-EF1 and its endolysin. PLoS One 8:e80435. doi:10.1371/journal.pone.0080435. 24236180 PMC3827423

[37] Lee D, Im J, Na H, Ryu S, Yun C-H, Han SH. 2019. The novel *Enterococcus* phagevB_EfaS_HEf13 has broad lytic activity against clinical isolates of *Enterococcus faecalis*. Front Microbiol 10:2877. doi:10.3389/fmicb.2019.02877. 31921055 PMC6927925

[38] Yang D, Chen Y, Sun E, Hua L, Peng Z, Wu B. 2020. Characterization of a lytic bacteriophage vB_EfaS_PHB08 harboring endolysin Lys08 against *Enterococcus faecalis* biofilms. Microorganisms 8:1332. doi:10.3390/microorganisms8091332. 32878334 PMC7564645

[39] De Klerk HC, Coetzee JN, Theron JJ. 1963. The characterization of a series of *Lactobacillus* bacteriophages. J Gen Microbiol 32:61–67. doi:10.1099/00221287-32-1-61. 14045524

[40] Lu Z, Breidt F, Fleming HP, Altermann E, Klaenhammer TR. 2003. Isolation and characterization of a *Lactobacillus plantarum* bacteriophage, ΦJL-1, from a cucumber fermentation. Int J Food Microbiol 84:225–235. doi:10.1016/s0168-1605(03)00111-9. 12781945

[41] Quiberoni A, Guglielmotti D, Binetti A, Reinheimer J. 2004. Characterization of three *Lactobacillus delbrueckii* subsp. *bulgaricus* phages and the physicochemical analysis of phage adsorption. J Appl Microbiol 96:340–351. doi:10.1046/j.1365-2672.2003.02147.x. 14723695

[42] Nabergoj D, Modic P, Podgornik A. 2018. Effect of bacterial growth rate on bacteriophage population growth rate. MicrobiologyOpen 7:e00558. doi:10.1002/mbo3.558. 29195013 PMC5911998

[43] Abedon ST. 2011. Lysis from without. Bacteriophage 1:46–49. doi:10.4161/bact.1.1.13980. 21687534 PMC3109453

[44] Kropinski AM, Mazzocco A, Waddell TE, Lingohr E, Johnson RP. 2009. Enumeration of bacteriophages by double agar overlay plaque assay, p 69–76. *In* Clokie MRJ, Kropinski AM (ed), Bacteriophages: methods and protocols, volume 1: isolation, characterization, and interactions. Humana Press, Totowa, NJ. 10.1007/978-1-60327-164-6_7 19066811

[45] Kropinski AM. 2009. Measurement of the rate of attachment of bacteriophage to cells, p 151–155. *In* Clokie MRJ, Kropinski AM (ed), Bacteriophages: methods and protocols, volume 1: isolation, characterization, and interactions. Humana Press, Totowa, NJ.

[46] Kutter E. 2009. Phage host range and efficiency of plating, p 141–149. *In* Clokie MRJ, Kropinski AM (ed), Bacteriophages: methods and protocols, volume 1: isolation, characterization, and interactions. Humana Press, Totowa, NJ. 10.1007/978-1-60327-164-6_14 19066818

[47] Worobo RW, Van Belkum MJ, Sailer M, Roy KL, Vederas JC, Stiles ME. 1995. A signal peptide secretion-dependent bacteriocin from *Carnobacterium divergens*. J Bacteriol 177:3143–3149. doi:10.1128/jb.177.11.3143-3149.1995. 7768812 PMC177004

[48] Besemer J, Lomsadze A, Borodovsky M. 2001. GeneMarkS: a self-training method for prediction of gene starts in microbial genomes. Implications for finding sequence motifs in regulatory regions. Nucleic Acids Res 29:2607–2618. doi:10.1093/nar/29.12.2607. 11410670 PMC55746

[49] McNair K, Zhou C, Dinsdale EA, Souza B, Edwards RA. 2019. PHANOTATE: a novel approach to gene identification in phage genomes. Bioinformatics 35:4537–4542. doi:10.1093/bioinformatics/btz265. 31329826 PMC6853651

[50] Ahn C, Stiles ME. 1990. Plasmid-associated bacteriocin production by a strain of *Carnobacterium piscicola* from meat. Appl Environ Microbiol 56:2503–2510. doi:10.1128/aem.56.8.2503-2510.1990. 2403256 PMC184756

[51] Zhang P, Badoni M, Gänzle M, Yang X. 2018. Growth of *Carnobacterium* spp. isolated from chilled vacuum-packaged meat under relevant acidic conditions. Int J Food Microbiol 286:120–127. doi:10.1016/j.ijfoodmicro.2018.07.032. 30081251

[52] Tulini FL, Lohans CT, Bordon KCF, Zheng J, Arantes EC, Vederas JC, De Martinis ECP. 2014. Purification and characterization of antimicrobial peptides from fish isolate *Carnobacterium maltaromaticum* C2: carnobacteriocin X and carnolysins A1 and A2. Int J Food Microbiol 173:81–88. doi:10.1016/j.ijfoodmicro.2013.12.019. 24412962

[53] Alves VF, De Martinis ECP, Destro MT, Vogel BF, Gram L. 2005. Antilisterial activity of a *Carnobacterium piscicola* isolated from Brazilian smoked fish (Surubim [*Pseudoplatystoma* sp.]) and its activity against a persistent strain of *Listeria monocytogenes* isolated from surubim. J Food Prot 68:2068–2077. doi:10.4315/0362-028x-68.10.2068. 16245709

[54] Martin-Visscher LA, van Belkum MJ, Garneau-Tsodikova S, Whittal RM, Zheng J, McMullen LM, Vederas JC. 2008. Isolation and characterization of Carnocyclin A, a novel circular bacteriocin produced by *Carnobacterium maltaromaticum* UAL307. Appl Environ Microbiol 74:4756–4763. doi:10.1128/AEM.00817-08. 18552180 PMC2519327

[55] Gursky LJ, Martin NI, Derksen DJ, van Belkum MJ, Kaur K, Vederas JC, Stiles ME, McMullen LM. 2006. Production of piscicolin 126 by *Carnobacterium maltaromaticum* UAL26 is controlled by temperature and induction peptide concentration. Arch Microbiol 186:317–325. doi:10.1007/s00203-006-0147-z. 16927067

