# Supplementary material for: Characterization of Bacteriophage cd2, a Siphophage Infecting Carnobacterium divergens and a Representative Species of a New Genus of Phage
Source: Microbiol Spectr. 2023 Jul 17;11(4):e00973-23. doi: 10.1128/spectrum.00973-23 (PMC10434151; doi:10.1128/spectrum.00973-23)
Supplement: Supplemental file 1 — Supplemental material. Download spectrum.00973-23-s0001.docx, DOCX file, 1.3 MB [file spectrum.00973-23-s0001.docx]

**Supplemental Information**

**Characterization of bacteriophage cd2, a siphophage infecting *Carnobacterium divergens* and a representative species of a new genus of phage**

Angelle P. Britton^a^, Kaitlyn A. Visser^a^, Véronique M.A. Ongenae^b,c^, Peipei Zhang^d^, Heather Wassink^a^, Thomas A. Doerksen^a^, Catherine A. Welke^a^, Karlene H. Lynch^e^, Marco J. van Belkum^f^, Jonathan J. Dennis^e^, Xianqin Yang^d^, Dennis Claessen^b,c^, Ariane Briegel^b,c^, and Leah A. Martin-Visscher^a,#^

^a^ Department of Chemistry, The King’s University, 9125 50 St, Edmonton, Alberta, Canada T6B 2H3

^b^ Molecular Biotechnology, Institute of Biology, Leiden University, PO Box 9505, 2300 RA Leiden, The Netherlands

^c^ Centre for Microbial Cell Biology, Leiden University, Leiden, The Netherlands

^d^ Agriculture and Agri-Food Canada, 6000 C&E Trail, Lacombe, Alberta, Canada T4L 1W1

^e^ Department of Biological Sciences, University of Alberta, Edmonton, AB T6G 2E9, Canada

^f^ Department of Chemistry, University of Alberta, Edmonton, AB T6G 2E9, Canada

^#^ Corresponding author: Leah A. Martin-Visscher (leah.martinvisscher@kingsu.ca)

**Table of Contents**

| **Methods & Results** | | | |  | | | | | | | | | | | | | |  | | |
| --- | --- | --- | --- | --- | --- | --- | --- | --- | --- | --- | --- | --- | --- | --- | --- | --- | --- | --- | --- | --- |
| **Initial isolation of phage cd2** | | | ……………………………………………………… | | | | | | | | | | | | | p. 1 | | |  |  |
| **Testing for lysogeny with PCR** | | | | …………………………………………………… | | | | | | | | | | | | p. 1 | | |  |  |
| **Genome sequences of *C. divergens* isolates** | | | | | | ………………………………………… | | | | | | | | | | p. 1 | | |  |  |
| **Phylogenetic analysis of phage sensitive *C. divergens* isolates** | | | | | | | | | | | …………………… | | | | | p. 1-2 | | |  |  |
| **Figures & Tables** | | | | | | | | | | | | | | | | | | |  |  |
| **Figure S1.** 1% agarose gel illustrating the results of the PCR lysogeny test | | | | | | | | | | | | | | …………. | | | p. 3 | | |  |
| **Figure S2.** Optimal MOI for phage cd2 | | | | | | ……………………………………………… | | | | | | | | | | | p. 4 | | |  |
| **Figure S3.** Intergenomic similarities heatmap for phage cd2 | | | | | | | | | ………………………… | | | | | | | | p. 5 | | |  |
| **Table S1.** Genome characteristics of *C. divergens* LV13 | | | | | | | | …………………………… | | | | | | | | | p. 6 | | |  |
| **Figure S4.** Maximum likelihood tree based on the core  genes of *C. divergens* isolates and *C. maltaromaticum* LMA28 | | | | | | | | | | | ……………………… | | | | | | p. 7 | | |  |
| **Table S2.** The orthologous average nucleotide identity (OrthoANI)  between *C. divergens* LV13 and other *C. divergens* isolates in this study | | | | | | | | | | | | | …………… | | | | p. 8 | | |  |
| **Table S3.** The pairwise core SNP distance of  *C. divergens* isolates included in this study (a mirrored table) | | | | | | | | | | ……………………… | | | | | | *Provided as Excel document* | | | |  |
| **Table S4.** Names and accession numbers for select  bacteriophages belonging to genus *Homburgvirus* and genus *Saphexaviru*s | | | | | | | | | | | | | | | ………… | | p. 9 | | |  |
| **References** | | | ……………………………………………………………………………… | | | | | | | | | | | | | | | p. 10-13 | | |

**Methods & Results**

**Initial isolation of phage cd2.**

Bacteriophage cd2 was initially isolated from the culture broth of a sample of minced beef, purchased at a grocery store in Edmonton, Alberta, Canada. A 1 g sample of meat was macerated in 10 mL of Brain Heart Infusion (BHI) broth and incubated overnight. Following centrifugation (9000 × *g*, 5 min, 4°C), the supernatant was filter-sterilized (0.2 μm) and 100 μL was spotted onto the center of a BHI agar plate that had been overlaid with soft agar containing 100 μL of an overnight culture of *Carnobacterium divergens* LV13 (1). After incubating for 16-18 h at 4°C, the clear zone of lysis was excised from the plate and mixed with 5 mL of BHI broth for 2-4 h (4°C). The mixture was centrifuged (9000 × *g*, 5 min, 4°C), after which the supernatant was filter sterilized (0.2 μm), serially diluted using BHI broth, and assessed for individual plaques using the double agar overlay method (2), using *C. divergens* LV13 as the indicator strain.

**Testing for lysogeny with PCR**

A PCR screening assay was performed to determine if phage cd2 was either lysogenic or pseudo-lysogenic in *C. divergens* B1, LV13 and C13. Phage cd2 lysate (10^9^ pfu/mL) was serially diluted and 5 μL of each sample was spotted onto an agar plate that had been overlaid with 5 mL of soft agar containing 100 μL of an overnight culture of strains B1, LV13 or C13, and the plate was incubated overnight at 25°C. To isolate potential lysogens, a sterile loop was rubbed over the centre of either turbid spots (for B1 and C13) or clear spots (for LV13) and then streaked onto a fresh BHI agar plate. After incubating the plates overnight at 25°C, individual colonies were subcultured and genomic DNA was extracted using the Qiagen DNeasy Blood and Tissue Kit according to manufacturer’s instructions. To serve as negative controls, genomic DNA was isolated from strains B1, LV13 and C13 that had not been exposed to phage cd2. To detect for the presence of phage DNA, PCR was performed using One*Taq* Quick-Load 2X Master Mix (New England Biolabs) and with primers CD2-A-F (5’-CCTCGTAAAGCTGATCCAACTG-3’) and CD2-A-R (5’-GTCTCCATAATACTGGAAGAACTC-3’), which were designed to amplify a region of phage cd2’s genome that included a portion of the terminase small subunit (amplicon size 935 bp). To confirm the presence of bacterial DNA, PCR was performed using primers B1-F (5’-CTCGCAATTATCGGATGGCC-3’) and B1-R (5’-TAGGTGCGGGAGCAGTGGT-3’), which are specific for the B1, LV13 and C13 bacterial genomes (amplicon size 1075 bp). Thermocycling conditions followed manufacturer’s instructions. PCR products were separated by agarose gel electrophoresis, stained with ethidium bromide, and visualized using a UV transilluminator (312 nm).

PCR results (illustrated in Figure S1) reveal that phage cd2’s genome was present in the DNA isolated from phage-exposed host strains B1 and LV13, but not in the phage-exposed host strain C13 host. Phage cd2’s genome was not present in the host strains if they had not been exposed to phage cd2. This suggests that phage cd2 is capable of lysogeny or pseudo-lysogeny in hosts B1 and LV13.

**Genome sequence of *C. divergens* isolates.**

The genomes of *C. divergens* isolates A2, A4, A8, A9-A13, B1-B18 and C1-C18 have previously been reported (3) and were downloaded from GenBank, while the genome of LV13 was sequenced in this study, as follows. Genomic DNA from *C. divergens* LV13 was extracted using the Qiagen DNeasy Blood and Tissue Kit according to manufacturer’s instructions. The sequencing library was constructed using Nextera® XT DNA library prep kit and sequenced using Illumina MiSeq PE250 platform with coverage >100. The quality of raw and trimmed sequencing reads was examined using FastQC v0.11.9 (4). Trimmomatic v0.39 (5) was used to remove adapters and reads with length <100 or average quality score in a 4-base sliding window < 30. The genome was assembled using SPAdes v3.14.0 (6) with the *k-*mers set at 21, 33, 55, 77, 99 and 127 bp. The quality of assembly was examined using Quast v5.0.2 (7). The assembled genome was filtered to remove contigs with coverage <10 or length < 500bp (8) and ordered using Mauve v2015-02-26 (9) with the genome of *C. divergens* DSM20623 (GenBank accession no. ASM74425v1) as a reference.

The size of assembled genome was about 2,762,513 bp and the GC content was 35.35% (Table S1). Sequencing data for LV13 is available in GenBank under BioProject number PRJNA851426. The Whole Genome Shotgun project has been deposited in GenBank under the accession number JAMXLU000000000. The version described in this paper is version JAMXLU010000000.

**Phylogenetic analysis of phage sensitive *C. divergens* isolates.**

To investigate the phylogenetic position of phage cd2 sensitive isolates, the genomes of all the *C. divergens* isolates used in this study were annotated using Prokka v1.14.6 (10). The pan-genome was parsed using Roary v3.13.0 with the identity threshold set at 90% (11). The phylogenetic tree was constructed using RaxML v8.2.12 (12) based on the concatenated core genes shared by all *C. divergens* isolates and *Carnobacterium maltaromaticum* LMA28 (13), the latter of which was used to root the tree. For RaxML, the general time reversible gamma nucleotide model (GTRGAMMA) was used, and bootstrap analysis was run for 100 times. To display the tree, ggtree package (14) in R was used. The orthologous average nucleotide identity (OrthoANI) among *C. divergens* isolates except for LV13 has previously been reported (15) and the same method was used to calculate the OrthoANI between LV13 and the other *C. divergens* isolates. Core single-nucleotide polymorphism (SNP) analysis was performed using Snippy v4.4.0 (<https://github.com/tseemann/snippy>) with the genome of *C. divergens* A10 as a reference.

Previously, it has been shown that genomes of *C. divergens* isolates A2, A4, A8, A9-A13, B1-B18 and C1-C18 can be separated into eight phylogenetic groups (IV-XI), where groups IV-VII formed one cluster while groups VIII-XI form another cluster (3, 15). The two clusters were found to have an orthologous average nucleotide identity (OrthoANI) of 93-94% which was lower than the threshold for species delineation (95-96%) and hence have been recommended to be classified as two distinct subspecies (15). Our phylogenetic analysis showed that *C. divergens* LV13 clusters with group VI isolates (Fig. S4). Consistently, LV13 shared the largest OrthoANI with group VI (mean, 99.99%) followed by group V (99.15%), IV (99.05%), VII (99.04%), XI (93.57%), VIII (93.56%), IX (93.54%) and X (93.47%) (Table S2).

Core SNP analysis was performed with isolate A10 as a reference. A total of 69654 SNP sites were found among the *C. divergens* isolates. The pairwise core SNP distance within each phylogenetic group and within each subspecies was ≤ 29 and 9992, respectively (Table S3, provided as an excel document). The genetic distance between two subspecies was between 55294 and 55681 SNPs. These results indicate a substantial amount of genetic variation between the two subspecies, hence, there is no obvious genetic distinction that can be linked to sensitivity or resistance to phage cd2. **Figures & Tables**

**
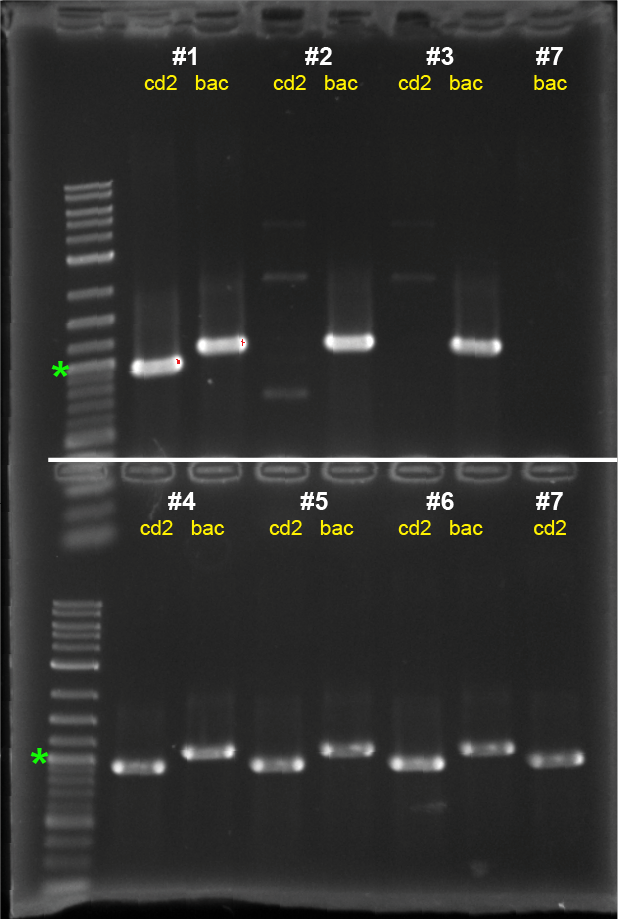
**

**Figure S1.** 1% agarose gel illustrating the results of the PCR lysogeny test. The template DNA used in each reaction is written in white text as follows: #1 is genomic DNA isolated from phage-exposed LV13, #2 is genomic DNA isolated from phage-exposed C13, #3 is genomic DNA isolated from wild-type B1 (not phage-exposed), #4 through #6 is genomic DNA isolated from phage-exposed B1(three different samples), and #7 is purified phage cd2 DNA. The primer pairs used in each reaction are written in yellow text, where “cd2” represents primers specific for phage cd2 (CD2-A-F/CD2-A-R) and “bac” indicates primer pairs specific for the host (B1-F/B1-R). The green asterisk shows the position of a 1000 bp band, and the bands directly above and below show the positions of 1200 bp and 900 bp bands, respectively. An amplicon of 935 bp indicates the presence of phage cd2 DNA, and an amplicon of 1075 bp indicates the presence of host DNA. These results show that phage cd2’s genome is present in DNA isolated from phage-exposed hosts LV13 and B1 (#1, #4 - #6), but not C13 (#2). Phage cd2’s genome was not present in DNA isolated from wild type (non-phage exposed) B1 (#3), or wild-type LV13 and C13 (data not shown). As expected, purified phage cd2 DNA gives a positive PCR result with the cd2 primers (#7, cd2) and a negative result with the host primers (#7, bac).

**
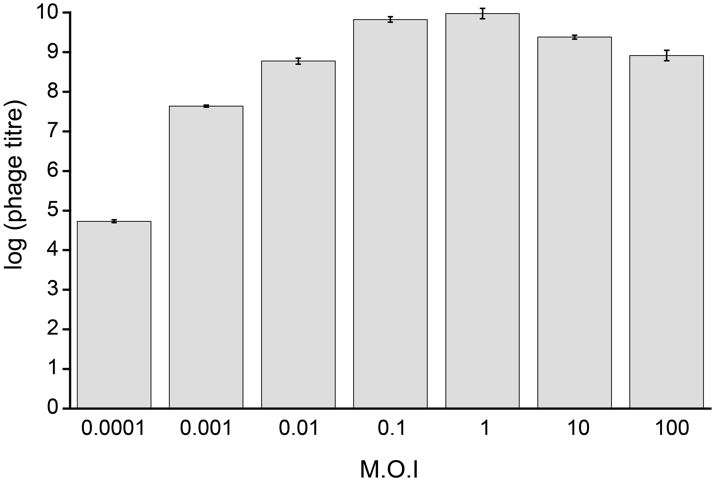
**

**Figure S2.** Optimal MOI for phage cd2. Mid-exponential phase cultures of *C. divergens* LV13 were infected with phage cd2, at various MOIs, for 10 min. After removing unadsorbed phage, the infected cells were incubated for 4 h and then assessed for total phage titre. The highest production of phage progeny was obtained when an initial MOI of 1.0 was applied. Therefore, the optimal MOI for phage cd2 is 1.0.


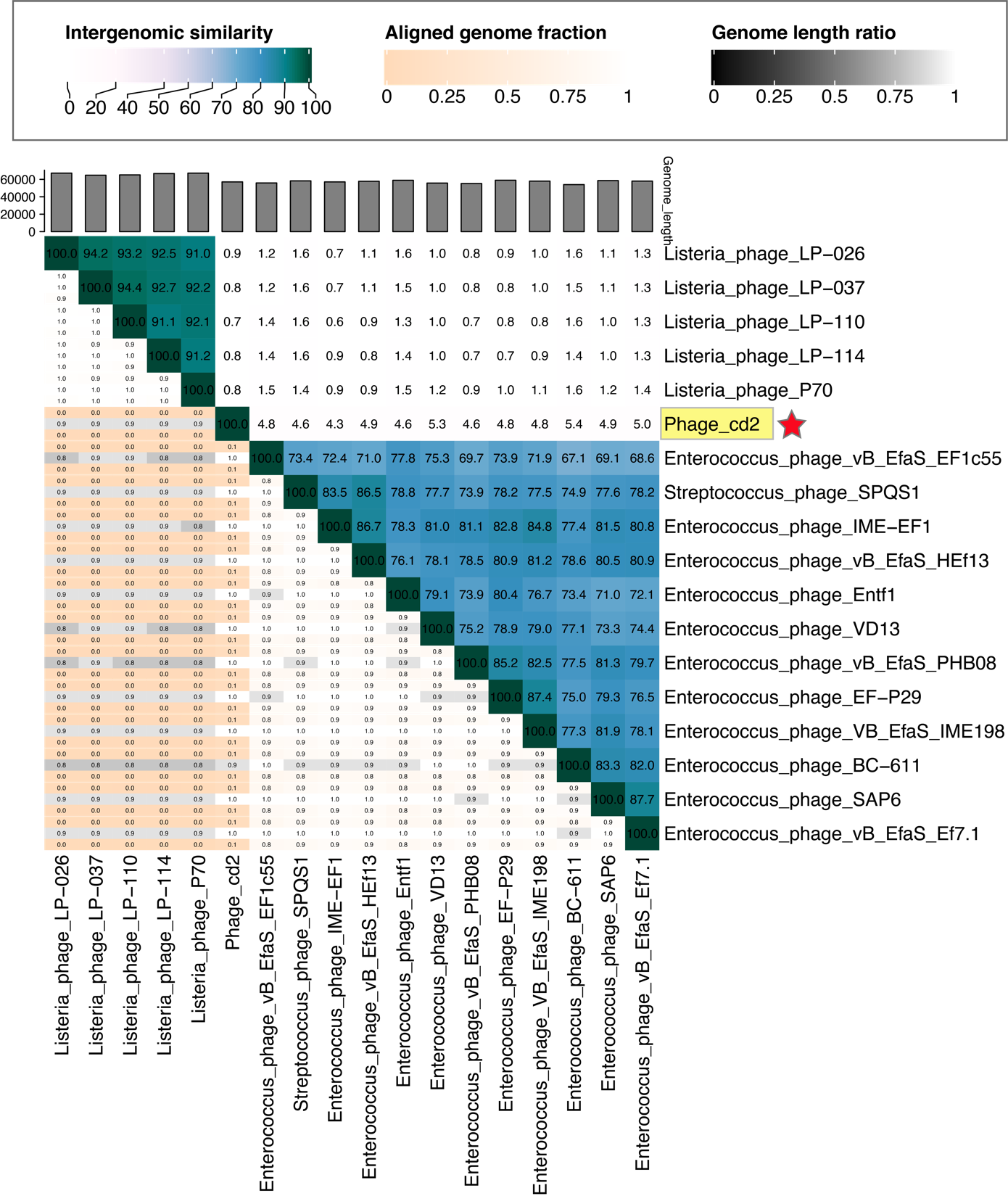


**Figure S3.** Intergenomic similarities heatmap generated with VIRIDIC, using default settings (17). The results suggest that *Carnobacterium* phage cd2 resides in a new phage genus since it has < 70% similarity to the *Listeria* phages (LP-037, LP-110, LP-114, P70), *Enterococcus* phages (vB_EfaS_EF1c55, IME-EF1, vB_EfaS_HEf13, Entf1, vB_EfaS_PHB08, EF-P29, vB_EfaS_IME198, VD13, BC611, SAP6, vB_EfaS_Ef7.1) and *Streptococcus* phage SPQS1. The position of phage cd2 is indicated by a red star and yellow box. Accession numbers and references for the phage genomes used in this analysis are provided in Table S4.

**Table S1.** Genome characteristics of *C. divergens* LV13.

| Total no. of raw reads | Approximate coverage by trimmed reads (x) | Assembled genome size (bp) | GC content (%) | No. of contigs | *N_50_* | No. of genes | GenBank accession no. | |
| --- | --- | --- | --- | --- | --- | --- | --- | --- |
|  |  |  |  |  |  |  | SRA | WGS assembly |
| 5539080 | 689 | 2762513 | 35.35 | 20 | 391475 | 2717 | [PRJNA851426](https://www.ncbi.nlm.nih.gov/sra/?term=PRJNA851426) | [JAMXLU000000000](https://www.ncbi.nlm.nih.gov/nuccore/JAMXLU000000000) |

**
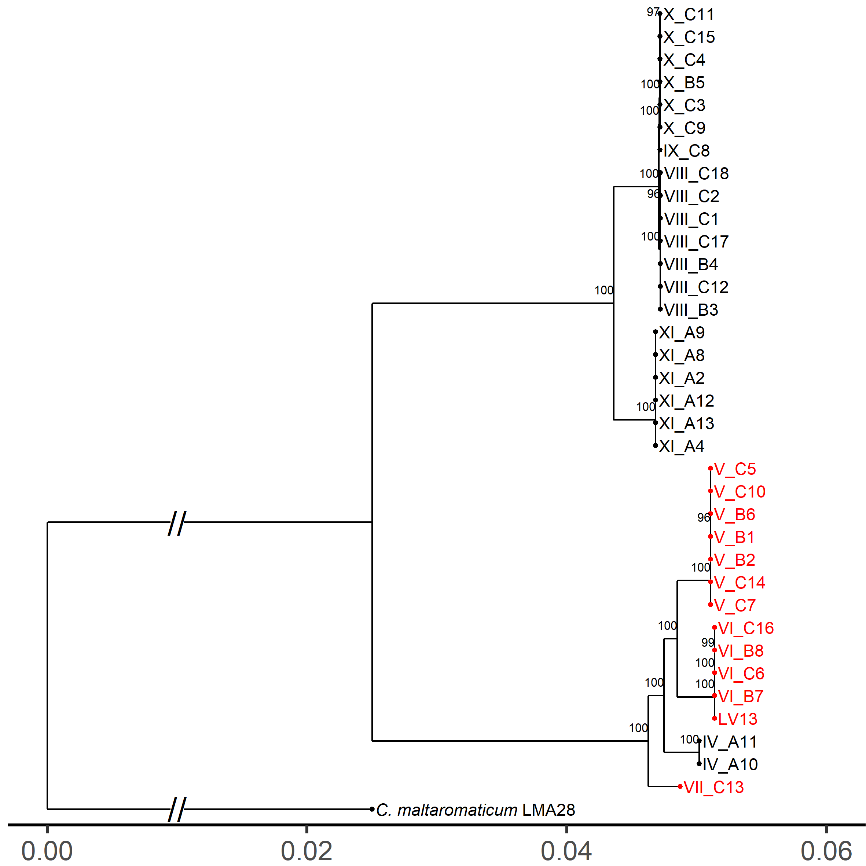
**

**Figure S4.** A maximum likelihood tree based on the core genes (n=732) of 35 *C. divergens* isolates and *C. maltaromaticum* LMA28 (outgroup). The scale bar represents the number of substitutions per site. The branch for outgroup isolate was truncated to show a higher resolution of the right side of the tree. The bootstrap value larger than 70 is shown on each node. All the isolates except for *C. divergens* LV13 were previously separated into eight phylogenetic groups (3, 15). Strains that are sensitive and resistant to phage cd2 are printed in red and black type, respectively. Strain names are identified by their phylogenetic group and isolate ID following the format “group_isolate id”, except for *C. divergens* LV13 and *C. maltaromaticum* LMA28, which are listed as such.

**Table S2.** The orthologous average nucleotide identity (OrthoANI) between *C. divergens* LV13 and the other *C. divergens* isolates in this study.

| **Phylogenetic**  **group** | **Isolate** | **OrthoANI (%)** | **Average OrthoANI**  **for each group (%)** |
| --- | --- | --- | --- |
| VI | C6 | 99.99 | 99.99 |
| VI | C16 | 99.99 |  |
| VI | B7 | 99.99 |  |
| VI | B8 | 99.99 |  |
| V | C10 | 99.16 | 99.15 |
| V | B6 | 99.15 |  |
| V | C14 | 99.15 |  |
| V | B2 | 99.15 |  |
| V | C7 | 99.15 |  |
| V | C5 | 99.15 |  |
| V | B1 | 99.14 |  |
| IV | A10 | 99.06 | 99.05 |
| IV | A11 | 99.05 |  |
| VII | C13 | 99.04 | 99.04 |
| XI | A8 | 93.62 | 93.57 |
| XI | A2 | 93.58 |  |
| XI | A13 | 93.54 |  |
| XI | A12 | 93.53 |  |
| XI | A4 | 93.52 |  |
| XI | A9 | 93.52 |  |
| VIII | B4 | 93.56 | 93.56 |
| VIII | C12 | 93.56 |  |
| VIII | C1 | 93.55 |  |
| VIII | C2 | 93.55 |  |
| VIII | C18 | 93.55 |  |
| VIII | B3 | 93.55 |  |
| VIII | C17 | 93.54 |  |
| IX | C8 | 93.54 | 93.54 |
| X | C15 | 93.48 | 93.47 |
| X | C3 | 93.48 |  |
| X | C11 | 93.47 |  |
| X | C4 | 93.47 |  |
| X | B5 | 93.47 |  |
| X | C9 | 93.45 |  |

**Table S4.** Names and accession numbers for select bacteriophages belonging to genus *Homburgvirus* and genus *Saphexaviru*s, along with phage cd2.

| **Phage name** | **Host** | **Genus** | **Accession no** | **Reference ^a^** |
| --- | --- | --- | --- | --- |
| Phage cd2 | *Carnobacteria* |  | MZ398135 | (18) |
| LP-026 | *Listeria* | *Homburgvirus* | NC_024375 | (19) |
| LP-037 | *Listeria* | *Homburgvirus* | NC_021787 | (19) |
| LP-110 | *Listeria* | *Homburgvirus* | NC_021785 | (19) |
| LP-114 | *Listeria* | *Homburgvirus* | NC_024392 | (19) |
| P70 | *Listeria* | *Homburgvirus* | NC_018831 | (20) |
| SPQS1 | *Streptococcus* | *Saphexaviru*s | NC_021868 | d.s. |
| VD13 | *Enterococcus* | *Saphexaviru*s | NC_024212 | (19) |
| BC611 | *Enterococcus* | *Saphexaviru*s | NC_018086 | (21) |
| SAP6 | *Enterococcus* | *Saphexaviru*s | NC_041960 | (22) |
| Entf1 | *Enterococcus* | *Saphexaviru*s | MK800154 | (23) |
| EF-P29 | *Enterococcus* | *Saphexaviru*s | KY303907 | (24) |
| vB_EfaS_EF1c55 | *Enterococcus* | *Saphexaviru*s | MN103542 | d.s. |
| IME-EF1 | *Enterococcus* | *Saphexaviru*s | NC_041959 | (23) |
| vB_EfaS_HEf13 | *Enterococcus* | *Saphexaviru*s | MH618488 | (25) |
| vB_EfaS_PHB08 | *Enterococcus* | *Saphexaviru*s | MK570225 | (26) |
| vB_EfaS_IME198 | *Enterococcus* | *Saphexaviru*s | NC_029016 | d.s. |
| vB_EfaS_Ef7.1 | *Enterococcus* | *Saphexaviru*s | MK721194 | d.s. |

**^a^** d.s. indicates direct submission of genome to GenBank

**References**

1. Ahn C, Stiles ME. 1990. Plasmid-associated bacteriocin production by a strain of *Carnobacterium piscicola* from meat. Appl Environ Microbiol 56:2503–2510.

2. Kropinski AM, Mazzocco A, Waddell TE, Lingohr E, Johnson RP. 2009. Enumeration of Bacteriophages by Double Agar Overlay Plaque Assay, p. 69–76. *In* Clokie MRJ, Kropinski AM (eds.), Bacteriophages: Methods and Protocols. Humana Press, Totowa, NJ.

3. Zhang P, Badoni M, Gänzle M, Yang X. 2018. Growth of *Carnobacterium* spp. isolated from chilled vacuum-packaged meat under relevant acidic conditions. Int J Food Microbiol 286:120–127.

4. Andrews S. 2010. FastQC: a quality control tool for high throughput sequence data. Available online: https://www.bioinformatics.babraham.ac.uk/projects/fastqc/

5. Bolger AM, Lohse M, Usadel B. 2014. Trimmomatic: a flexible trimmer for Illumina sequence data. Bioinformatics 30:2114–2120.

6. Bankevich A, Nurk S, Antipov D, Gurevich AA, Dvorkin M, Kulikov AS, Lesin VM, Nikolenko SI, Pham S, Prjibelski AD, Pyshkin AV, Sirotkin AV, Vyahhi N, Tesler G, Alekseyev MA, Pevzner PA. 2012. SPAdes: A New Genome Assembly Algorithm and Its Applications to Single-Cell Sequencing. J Comput Biol 19:455–477.

7. Gurevich A, Saveliev V, Vyahhi N, Tesler G. 2013. QUAST: quality assessment tool for genome assemblies. Bioinformatics 29:1072–1075.

8. Douglass AP, O’Brien CE, Offei B, Coughlan AY, Ortiz-Merino RA, Butler G, Byrne KP, Wolfe KH. 2019. Coverage-Versus-Length Plots, a Simple Quality Control Step for *de Novo* Yeast Genome Sequence Assemblies. G3 879–887.

9. Darling ACE, Mau B, Blattner FR, Perna NT. 2004. Mauve: Multiple Alignment of Conserved Genomic Sequence With Rearrangements. Genome Res 14:1394–1403.

10. Seemann T. 2014. Prokka: rapid prokaryotic genome annotation. Bioinformatics 30:2068–2069.

11. Page AJ, Cummins CA, Hunt M, Wong VK, Reuter S, Holden MTG, Fookes M, Falush D, Keane JA, Parkhill J. 2015. Roary: rapid large-scale prokaryote pan genome analysis. Bioinformatics 31:3691–3693.

12. Stamatakis A. 2014. RAxML version 8: a tool for phylogenetic analysis and post-analysis of large phylogenies. Bioinformatics 30:1312–1313.

13. Cailliez-Grimal C, Chaillou S, Anba-Mondoloni J, Loux V, Afzal MI, Rahman A, Kergourlay G, Champomier-Vergès M-C, Zagorec M, Dalgaard P, Leisner JJ, Prévost H, Revol-Junelles A-M, Borges F. 2013. Complete Chromosome Sequence of *Carnobacterium maltaromaticum* LMA 28. Genome Announc 1:e00115-12.

14. Yu G. 2020. Using ggtree to Visualize Data on Tree-Like Structures. Curr Protoc Bioinforma 69:e96.

15. Zhang P, Gänzle M, Yang X. 2019. Complementary Antibacterial Effects of Bacteriocins and Organic Acids as Revealed by Comparative Analysis of *Carnobacterium* spp. from Meat. Appl Environ Microbiol 85:e01227-19.

16. Nishimura Y, Yoshida T, Kuronishi M, Uehara H, Ogata H, Goto S. 2017. ViPTree: the viral proteomic tree server. Bioinformatics 33:2379–2380.

17. Moraru C, Varsani A, Kropinski AM. 2020. VIRIDIC-A Novel Tool to Calculate the Intergenomic Similarities of Prokaryote-Infecting Viruses. Viruses 12:1268.

18. Zhang P, Britton AP, Visser KA, Welke CA, Wassink H, Prins E, Yang X, Martin-Visscher LA. 2021. Genome Sequences of Bacteriophages cd2, cd3, and cd4, which Specifically Target *Carnobacterium divergens*. Microbiol Resour Announc 10:e00636-21.

19. Denes T, Vongkamjan K, Ackermann H-W, Moreno Switt AI, Wiedmann M, den Bakker HC. 2014. Comparative Genomic and Morphological Analyses of *Listeria* Phages Isolated from Farm Environments. Appl Environ Microbiol 80:4616–4625.

20. Schmuki MM, Erne D, Loessner MJ, Klumpp J. 2012. Bacteriophage P70: Unique Morphology and Unrelatedness to Other *Listeria* Bacteriophages. J Virol 86:13099–13102.

21. Horiuchi T, Sakka M, Hayashi A, Shimada T, Kimura T, Sakka K. 2012. Complete Genome Sequence of Bacteriophage BC-611 Specifically Infecting *Enterococcus faecalis* Strain NP-10011. J Virol 86:9538–9539.

22. Lee Y-D, Park J-H. 2012. Complete Genome Sequence of Enterococcal Bacteriophage SAP6. J Virol 86:5402–5403.

23. Zhang W, Mi Z, Yin X, Fan H, An X, Zhang Z, Chen J, Tong Y. 2013. Characterization of *Enterococcus faecalis* Phage IME-EF1 and Its Endolysin. PLoS ONE 8:e80435.

24. Cheng M, Liang J, Zhang Y, Hu L, Gong P, Cai R, Zhang L, Zhang H, Ge J, Ji Y, Guo Z, Feng X, Sun C, Yang Y, Lei L, Han W, Gu J. 2017. The Bacteriophage EF-P29 Efficiently Protects against Lethal Vancomycin-Resistant *Enterococcus faecalis* and Alleviates Gut Microbiota Imbalance in a Murine Bacteremia Model. Front Microbiol 8:837.

25. Lee D, Im J, Na H, Ryu S, Yun C-H, Han SH. 2019. The Novel *Enterococcus* Phage vB_EfaS_HEf13 Has Broad Lytic Activity Against Clinical Isolates of *Enterococcus faecalis*. Front Microbiol 10:2877.

26. Yang D, Chen Y, Sun E, Hua L, Peng Z, Wu B. 2020. Characterization of a Lytic Bacteriophage vB_EfaS_PHB08 Harboring Endolysin Lys08 against *Enterococcus faecalis* Biofilms. Microorganisms 8:1332.
